# Tumor-derived exosomes as predictors of chemotherapy resistance in gastric cancer

**DOI:** 10.1097/MS9.0000000000004415

**Published:** 2025-11-25

**Authors:** Muhammad Khizar, Hasibullah Aminpoor, Muhammad Zaib, Qaima Ali, Hasiba Karimi

**Affiliations:** a Faculty of Medicine, Georgian American University, Manifest Medical Research Co, Tbilisi, Georgia; b Faculty of Medicine, Kabul University of Medical Sciences “Abu Ali Ibn Sina”, Kabul, Afghanistan; c Faculty of Medicine, Liaquat College of Medicine & Dentistry, Karachi, Pakistan; d Faculty of Medicine, Bezmialem Vakif University, Istanbul, Turkey

**Keywords:** biomarkers, chemotherapy resistance, exosomal miRNAs, gastric cancer, liquid biopsy, personalized medicine, precision oncology, tumor-derived exosomes

## Abstract

Chemotherapy resistance is a major obstacle in gastric cancer, limiting the efficacy of systemic therapy and contributing to poor survival. Tumor-derived exosomes are key mediators of chemoresistance, carrying microRNAs, long noncoding RNAs, and proteins that modulate apoptosis, drug efflux, and tumor–microenvironment interactions. Evidence from high-incidence countries, such as China and Japan, associates specific exosomal signatures with poor treatment response, and translational studies in Europe and North America are exploring their prognostic and predictive utility. Despite technical advances, clinical translation is hampered by inconsistent protocols, small cohorts, and limited regulatory frameworks. This letter reviews the biological and clinical evidence for exosome-mediated chemoresistance in gastric cancer and advocates for standardized methodologies, global validation studies, and integration into precision oncology pathways.

*To the Editor,*

Chemotherapy resistance represents a major challenge in the management of gastric cancer, significantly limiting survival benefits and contributing to treatment failure. Tumor-derived exosomes (TDEs), nanoscale extracellular vesicles carrying nucleic acids, proteins, and lipids, have emerged as promising biomarkers capable of reflecting and potentially mediating chemoresistant phenotypes. Understanding their role offers an opportunity to improve patient stratification, optimize therapy, and advance precision oncology in gastric malignancy^[1,2]^.

Exosomes are formed within multivesicular bodies and released into the extracellular space, where they facilitate intercellular communication^[1]^. In gastric cancer, these vesicles modulate the tumor microenvironment, promote immune evasion, induce epithelial-mesenchymal transition, and influence metastatic niche formation^[3]^. Importantly, exosomes carry molecules that confer chemoresistance when transferred to recipient cells. For example, exosomal miR-21 and miR-155 downregulate tumor suppressor genes and activate drug efflux pathways, leading to resistance against cisplatin and fluoropyrimidine-based therapies^[2,4]^. Preclinical studies have also shown that exosomal proteins and RNAs can horizontally transfer resistance phenotypes across cell populations, highlighting their functional significance^[2,4]^.

Clinically, exosome profiling from liquid biopsies (blood, peritoneal fluid, or ascites) offers a noninvasive window into tumor biology. In China and Japan, elevated exosomal miRNAs correlate with advanced disease and poor prognosis^[5]^. In Japan, Makinoya *et al* demonstrated that exosomal miR-493 in peritoneal fluid mediates docetaxel and paclitaxel resistance by downregulating MAD2L1, directly linking exosomal content to chemotherapy response^[6]^. In Europe and North America, plasma exosomal PD-L1 and exosomal proteins are being evaluated as predictors of fluoropyrimidine and oxaliplatin resistance, suggesting their potential to identify non-responders early and guide therapeutic adjustments^[3]^. Integrating such biomarkers could spare patients from ineffective treatments and associated toxicity while enabling timely escalation or modification of therapy.

Technological innovations have further expanded the translational potential of exosomes. High-throughput mass spectrometry and next-generation sequencing allow detailed characterization of exosomal cargo, while experimental approaches targeting exosome biogenesis, for example via neutral sphingomyelinase inhibitors, restore chemosensitivity in resistant cell lines^[7]^. Multi-center initiatives in South Korea and Australia are exploring the feasibility of integrating exosomal panels with conventional markers such as CEA and CA19-9 to improve risk stratification and treatment selection^[1]^. These approaches emphasize the promise of precision medicine through dynamic monitoring of tumor biology.

Despite this promise, translation into routine practice remains limited. Many studies are single-center, include small cohorts, and lack diversity. Standardization of sample collection, exosome isolation, and quantification is insufficient, resulting in inconsistent reproducibility. For example, a tertiary center in Brazil attempted to stratify FOLFOX-treated patients using exosomal miRNA levels but observed unreliable predictive performance due to variable plasma processing and storage conditions^[4]^. Similarly, in India, heterogeneous pre-analytical methods hindered clinical validation of exosomal miRNAs in gastrointestinal cancers^[1]^. Addressing these challenges requires consensus protocols, multiethnic cohort validation, for example from China, Japan, and Turkey, and regulatory pathways to ensure clinical reliability and global applicability.

In conclusion, TDEs are promising biomarkers for predicting chemotherapy resistance in gastric cancer. They capture dynamic tumor biology noninvasively and may guide personalized treatment, optimize therapy, and reduce exposure to ineffective regimens. To translate these findings into practice, standardization, multicenter validation, and regulatory alignment are essential. With coordinated global efforts, exosome-based diagnostics could become an integral tool in precision-guided gastric cancer management.

Our work is in line with the TITAN Guidelines on the need for transparency in artificial intelligence use in healthcare^[7]^.

## Data Availability

Not applicable.
